# Small extracellular vesicles secreted by vaginal fibroblasts exert inhibitory effect in female stress urinary incontinence through regulating the function of fibroblasts

**DOI:** 10.1371/journal.pone.0249977

**Published:** 2021-04-09

**Authors:** Xiaoyan Sun, Huimin Zhu, Wenjuan Li, Li Zhao, Wenhua Li, Xiaoyong Li, Zhenwei Xie

**Affiliations:** Department of Gynecology, Women’s Hospital School of Medicine Zhejiang University, Hangzhou, Zhejiang, P.R. China; Xiangtan University, CHINA

## Abstract

Stress urinary incontinence (SUI) is a common condition in women and associated with extra-cellular matrix (ECM) reconstruction, which is mainly regulated by fibroblasts. However, the underlying mechanism remains obscure. Small extracellular vesicles (sEVs) play fundamental biological roles in various cellular functions. Some studies suggested that the sEVs were involved in the metabolism of ECM and the function of fibroblasts. The purpose of our study was to investigate the effect of sEVs secreted by vaginal fibroblasts on the pathogenesis of SUI. We showed that the fibroblasts of female anterior vaginal wall secreted sEVs. Moreover, fibroblasts of females with SUI had significantly elevated secretion of sEVs. The collagen contents, proliferation and migration capacity of fibroblasts were decreased when fibroblasts were co-cultured with fibroblasts-derived sEVs (fibroblast-sEVs) from SUI patients. Proteomic analysis revealed that fibroblast-sEVs contained various differentially expressed proteins including TIMP2, TGF-β and ABCC4, which were involved in signaling pathways of fibroblasts regulation. Therefore, we suggested that fibroblast-sEVs contributed to the pathogenesis of SUI through various proteins including TIMP2, TGF-β and ABCC4.

## Introduction

Stress urinary incontinence (SUI) is defined as the involuntary leakage of urine during laughing, coughing, sneezing or physical exercise. SUI affects about 61% of women and represents a significant social and economic burden [1, 2]. Moreover, as age increases, the leakage becomes worse and harder to be reversed. However, the pathogenesis of SUI remains unclear.

Many studies suggested that the development of SUI was accompanied by fibroblast dysfunction [3–6]. The bladder and urethra were kept in place by the anterior vaginal wall which consisted of a dense extra-cellular matrix (ECM) regulated by fibroblasts [6]. Fibroblasts remodeled their surrounding matrix and maintained tissue homeostasis by altering own functional activity and producing active substance, which was involved in soft tissue repair [6]. Some studies showed that increased apoptosis rate and altered metabolism of ECM (specifically collagen) in fibroblasts contributed to pathogenesis of SUI [3, 5]. And, as the effective treatment of SUI, electrical stimulation upregulated collagen expression of fibroblasts and exhibited a cell proliferation-promoting effect [4]. In addition, studies revealed that transurethral injections of autologous fibroblasts effectively improved the symptom of SUI, reconstructed the urethral submucosa and increased the thickness of rhabdosphincter in SUI patients [7, 8]. Therefore, the ECM reconstruction, which was regulated by fibroblasts, played an important role in the pathogenesis of SUI.

Intercellular communication of fibroblasts was closely associated with the metabolism of ECM. The sEVs (also known in the past as exosomes), the group of nanosized extracellular vesicles with a diameter of about < 200nm, were reported to be the key intercellular communication mediators between cells [9]. The sEVs delivered various biologically active substances to target cells and participated in numerous physiological and pathological processes [10]. Further investigations showed that as specific modulators of the ECM, sEVs regulated target cells to synthesize or degrade matrix molecules and participated in matrix organization. They had been considered as one of the structural and functional components of the ECM [11, 12]. The sEVs could be secreted by a variety of human tissue cells. And fibroblasts-derived sEVs (fibroblast-sEVs) had been proved to be involved in the biological processes of severe asthma, diabetic ulcers and cancers [13–15]. Meanwhile, fibroblast-sEVs regulated the ECM remodeling of focal tissues by changing the function of regional fibroblasts, stabilizing the self-polarization state, orienting the aggregation and regenerating the collagen fibers in the autocrine manner [16–18].

Therefore, the purpose of this study was to verify whether vaginal fibroblasts could secrete sEVs, to compare the function of sEVs from SUI patients (SUI-sEVs) and non-SUI patients (non SUI-sEVs). Moreover, we also attempted to investigate how sEVs affected the functions of fibroblasts, and further offered new insights to the possible mechanism in SUI.

## Materials and methods

### Ethics statement

With the informed consent of the patients and the approval by the Ethics Committee of Women’s Hospital School of Medicine Zhejiang University (Approval NO.20140040), human vaginal anterior wall tissues were collected between December 2017 and April 2019. Fibroblasts were isolated and cultured, some cells were cryopreserved and archived for follow-up study. And, with the approval by the Ethics Committee of Women’s Hospital School of Medicine Zhejiang University (Approval NO.20190073), above-mentioned archived fibroblasts were used for the current research from August 2019.

### Subjects and sample collection

SUI was diagnosed in accordance with the criterion of the International Continence Society [19].Five patients with SUI who underwent tension-free vaginal tape surgery at the Women’s Hospital of Medicine Zhejiang University between December 2017 and April 2019 were recruited. Three patients without SUI who underwent paraurethral cyst excision within the same time period were recruited as control subjects. All subjects had no history of malignant tumors and estrogen-dependent disease, and were free of urinary infection at time of surgery. Biopsy samples of the anterior vaginal wall were excised 2 cm above from the external urethral orifice.

### Cells culture

Isolation, identification and culture of fibroblasts were executed as described previously [20]. Isolated fibroblasts were cultured in the 37°C incubator with 5% CO_2_. The fibroblasts were passaged when the cells were 70% confluent, and cells of passage 3 were selected for subsequent experiments.

### Isolation of sEVs

The fibroblast-sEVs were extracted by ultracentrifugation as previously described [21]. Passage 3 cells were seeded into the 10ml sterile culture dish plate with a density of 1×10^6^ cells/ml. Serum-free D-MEM/F12 culture medium (1×) (culture medium: China Branch Barter No. COO12) was replaced to culture the cells for 48 h when the cell filling rate reached 80%. Then successive centrifuged the supernatants at 400g for 10 minutes and 2,000g for 10 min at 4°C to eliminate the cells and dead cells. Next, we filtered the centrifuged supernatants by the Millipore Millex-GP needle filter (Millipore Millex-GP 0.22um, USA) to eliminate the cell debris. The filtered supernatants were successively ultracentrifuged at 110,000g for 90 min at 4°C (cryogenic ultracentrifuge: Beckman, USA) to exclude the contaminant proteins, then the supernatants were thrown away and the pellets were used for the following experiment. Finally, the fibroblast-sEVs pellets were re-suspended in 50μl of PBS.

### Electron microscope analysis

The morphological characteristics of sEVs were observed by transmission electron microscopy (TEM) (FEI, FEI Tecnai T10, USA). Placed 10μl resuspended pellets on the copper grids and incubated at room temperature for 10 min. Fixed the pellets using moderate 2.5% glutaraldehyde at room temperature for 5 min. Washed the grids and negative stained the pellets on the grids with 2% uranium acetate for 5 min. Cleaned the grids and observed the samples under the transmission electron microscopy (TEM) (FEI, FEI Tecnai T10, USA) at 80 Kv.

### Particle size analysis

Particle size of sEVs were assessed by nanoparticle size tracking analyzer (NTA) (Malvern, NanoSight NS 300, England). An aliquot of 5μl sEVs solutions were diluted to 1ml with PBS. Filtered the mixture of the sEVs and PBS with 0.22μm filter membrane. The attenuated and filtered sEVs were detected by NTA. Statistical analyses were performed using NTA statistical software (Version NTA 3.2 Dev Build 3.2.16 NTA).

### BCA protein assay

An aliquot of 5μl sEVs resuspend solutions with the dry block heater (All for life science, K30, China) was heated at 95°C for 15 min. The protein concentrations of sEVs were measured by using the BCA protein assay reagent kit (Beyotime, P0010, China) according to the manufacturer’s instructions. The standard protein concentration curve was drawn by ultraviolet spectrophotometer, and the protein concentration of each sample was calculated.

### Western blot analysis

Western blot was performed in accordance with the protocol described previously [20].The proteins were loaded into prepared 10% SDS-PAGE gel, and transferred onto the 0.22-μm poly-vinylidene difluoride membranes (EMD Millipore, Billerica, MA,USA). Then blocked the membranes with corresponding blocking solution for 1 h and incubated with primary antibodies for 2 h. Subsequently, cleaned the membranes and incubated with secondary antibody for 1 h. Finally, re-cleaned the membranes and visualized the protein signals using FDbio-Femto enhanced chemiluminescence detection kit-HRP (Fdbio science, Cat No:FD8030, China) and chemiluminescence imager (Ge healthcare, Imagequant LAS 4000 Mini, USA). The data was analyzed using the imagequant TL software (Ge healthcare, USA). Primary antibodies were anti-CD63 antibody (1: 500, cat. ab59479, Abcam, UK), anti-CD9 antibody (1:2000, cat. ab92726, Abcam, UK), anti-collagen I antibody (1:1000, Santa Cruz Biotechnology, sc-59772, USA), and anti-collagen III antibody (1: 1000, Bioss, bs-0549R, China). The secondary antibodies were the goat anti-mouse IgG (H+L) secondary antibody (1:5000, cat.31160, Thermo Pierce, USA) and anti-rabbit IgG, HRP-linked antibody (1:2000, cat.7074, Cell Signaling Technology, USA). The anti-beta-actin antibody (1:5000; Santa Cruz Biotechnology, sc-59772, USA) was used for normalization of protein expression.

### sEVs co-cultured with primary fibroblasts

Co-culture of sEVs with fibroblasts were performed using the 24-well culture plate. Primary non-SUI fibroblasts were divided into S-sEVs group (co-culture of fibroblasts and fibroblast-sEVs from SUI subjects), N-sEVs group (co-culture of fibroblasts and fibroblast-sEVs from non-SUI subjects) and control group (co-culture of fibroblasts and culture medium). The 0.25μg/ml protein concentration of sEVs from SUI (SUI-sEVs) and non-SUI fibroblasts (non SUI-sEVs) were prepared and co-cultured with a density of 2 ×10^4^ cells/ml primary fibroblasts for the follow-up cell proliferation, migration, western blot experiments.

### Cell migration analysis

A horizontal line was drawn evenly in the center of the bottom of the plate with 10μl micropipette tip to mark the site before co-culture. Photographed the cells under optical microscope (10×20 times) to observe the cell migration of fibroblasts at 0, 24, and 48 h after the co-culture.

### Cell proliferation analysis

The cell counting kit-8 (CCK-8, Biosharp, BS350B, China) was used to assess the cell proliferation of cultured fibroblasts. According to the manufacturer’s instructions, cell proliferation was assayed on 0, 24, 48, and 72 h after the co-culture. An aliquot of 10ul of the reagent with Cell Counting Kit-8 were added to each well, and the plates were incubated at 37°C for 1 h. Finally, the absorbance was measured by Microplate Reader (iMarkTM; Bio-Rad, USA) at 450 nm.

### Proteomic analysis

#### Sample preparation

Lysis buffer (2% SDS, 7M Urea, Protease Inhibitor Cocktail) was added into the collected sEVs and cracked using the ultrasonic processor for 1min. Centrifuged the lysate at 13000rpm for 20 min at 4°C. Collected the supernatant, and acetone was added to a 6:1 ratio (acetone to supernatant). Incubated the mixture at -20°Covernight, then centrifuged to collect the protein pellet. Washed the pellet and redissolved it in buffer (300mM triethylamine borane, TEAB, 6 M guanidine hydrochloride), following by centrifuging 13000rpm for 10 min at 4°C. Then, quantitated the protein by the BCA assay. Digested protein was performed according to the filter-aided sample preparation (FASP) procedure. Briefly, determined the protein solution samples to 200ul with 25mM ammonium bicarbonate. Added 1 M DTT (terminal concentration 20mM) to incubate for 1 h at 57°C. Then, added 20ul iodoacetamide (terminal concentration 90mM) and incubated for 40 min at room temperature under dark conditions. Centrifuged the samples on the 10KDa ultrafiltration tubes at 12000rpm, and added ammonium bicarbonate to wash 4 times. Digested the samples with trypsin at 37°C overnight. Collected the peptides after centrifugation, and dried by centrifugal concentration. Desalted the peptides using the Ziptip C18 column.

#### Mass spectrometry analysis

Dissolved the dried samples in buffer (0.1% formic acid). The on-line Nano-RPLC liquid chromatography was performed by Easy-nLC 1200 system (Thermo Scientific). The mobile phases consisted of Solution A (0.1% formic acid) and Solution B (0.1% formic acid in 80%ACN). Peptides were trapped in 99% Solution A in the trap column (home-made C18, 5um, 100um×2cm). Separation was performed in C18 reversed-phase column (C18, 1.9um, 75um×200mm) at a flow rate of 200 nl/min and the gradient was as follows: 2–5% B in 2 min, 5–35% B in 100 min, 35–44% B in 6 min, 44–100% B in 3min, 100% B for 10min. The peptides results were subjected to nano electrospray ionization source followed by tandem mass spectrometry in Orbitrap Fusion Lumos (Thermo Fisher Scientific). The LC-MS analysis used data—dependent acquisition (DDA) pattern. The full sweep resolution was 60,000 (FWHM), the mass/charge ratio range was set to m/z 375–1600, and the collision energy was set to 30% in HCD fragmentation mode.

#### Biostatistical and informatics analysis

For database searching, the MaxQuant (version 1.5.8.3, Max-Planck Institute for Biochemistry, Germany) was used to search against the Swiss-Prot database (www.uniprot.org Octorber, 2019 release, 20,285 protein entries). Search for trypsin-digested peptides with 2 allowed missed cleavages permitted, and carbamidomethylation was defined as fixed modification, oxidation (M) and acetylation (N-terminal) were defined as variable modifications for database searches. PSM FDR was set at 0.01 calculated by the target-revert decoy approach. For the study of the DEP’s (different expression proteins) biological functions and to reveal which pathways were significantly represented by the DEP, the Gene Ontology (GO) analysis and the pathway analysis were performed using the database of DAVID 6.8 (available at https://david.ncifcrf.gov/).).

### Statistical analysis

Continuous variables were expressed as mean±SD. Statistical analysis was performed using GraphPad Prism 8.0 statistical software (GraphPad Software Inc., San Diego, CA). Means changes between groups were compared by independent samples Student’s t test. Chi-square test were used to compare the categorical data. *P*-values < 0.05 were considered statistically significant.

## Results

### Isolation and characterization of fibroblast-sEVs

The characteristics of fibroblast-sEVs had been published as a conference paper previously [22], manifesting the isolated particles had characteristic of fibroblast-sEVs which were suitable for following experiments.

### Elevated expression of fibroblast-sEVs in patients with SUI

**Fig 1:**
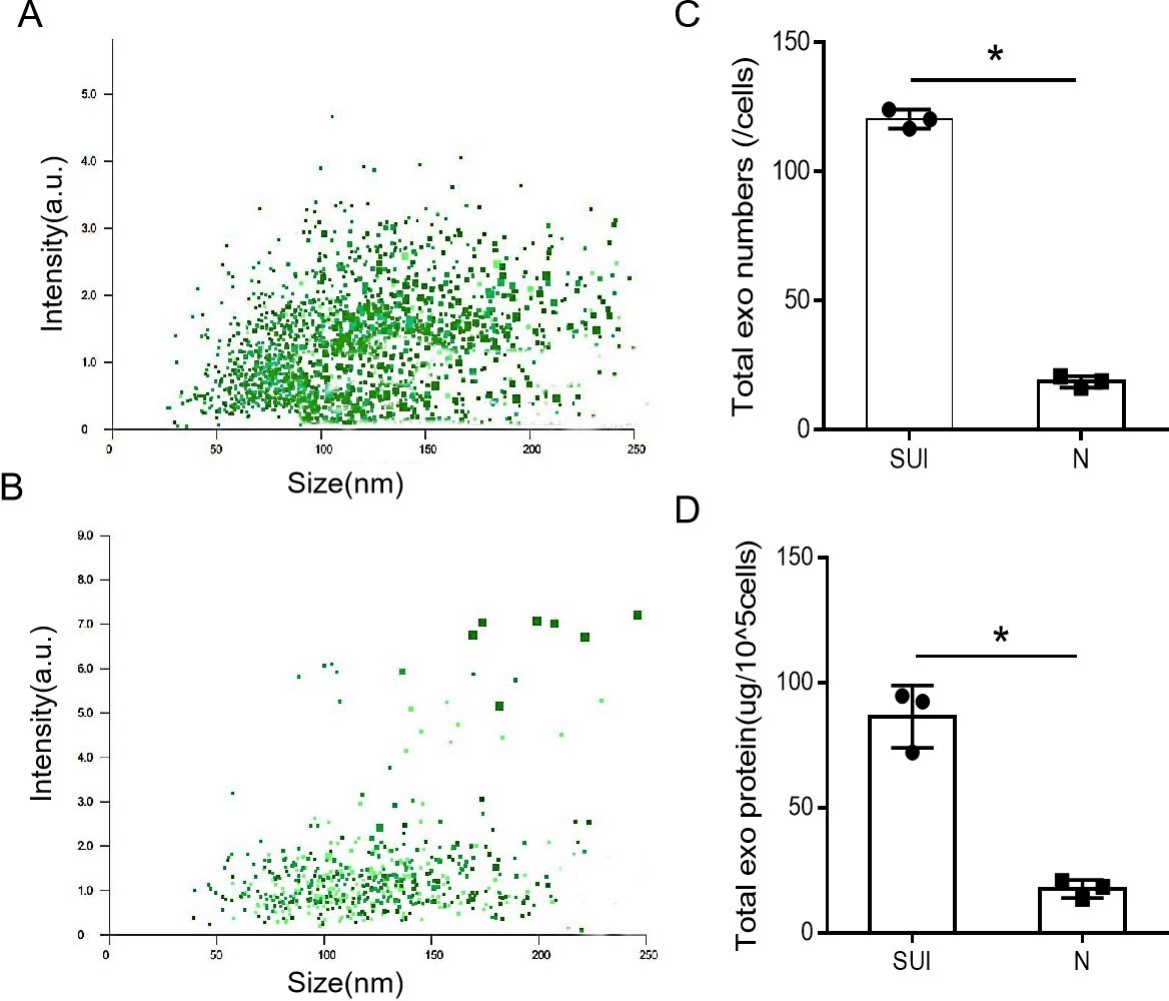
The fibroblast-sEVs from patients with SUI was elevated. (A and B) Scatter plot distribution of SUI-sEVs and non SUI-sEVs with Nanoparticle tracking analysis measurements. (C) Quantitative comparison results of the number of sEVs in the SUI and non-SUI groups. (D) Semi-quantitative comparison results of total protein content of sEVs in the SUI and non-SUI groups. N: non-SUI group, SUI: SUI group, N = 3 in each group. Data represented means±SD of 3 independent experiments. * t-test *P* value <0.05.

By Using BCA protein quantification and NTA methods, we evaluated the quantity of sEVs from subjects with SUI and non-SUI. The mean total protein content of sEVs from the certain number of fibroblasts (10^5^ cells) was 86.41±7.12ug in the SUI subjects, and 17.57±2.11ug in the non-SUI subjects, which was statistically significant (*P* = 0.008).The mean number of sEVs form single fibroblast was 120.30 ± 2.13 particles in the SUI subjects and 18.65 ± 1.25 particles in the non-SUI subjects, which was statistically significant *(P* < 0.0001). These results indicated that the quantity of fibroblast-sEVs was increased in patients with SUI (Fig 1).

### SUI-sEVs decreased collagen expression of fibroblasts

**Fig 2:**
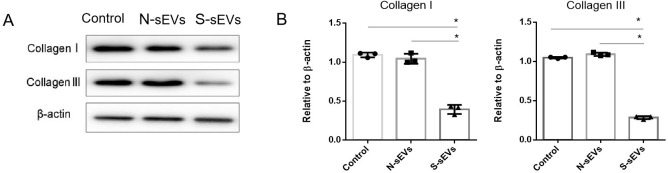
SUI-sEVs negatively regulated type I and III collagen expression in fibroblasts. Fibroblasts were incubated with control medium or medium containing SUI-sEVs (S-sEVs) or medium containing non SUI-sEVs (N-sEVs) for 48 h. (A) The expression of type I and III collagen were determined by the Western blot assay. (B) Quantification of type I and III collagen expression. N = 3 in each group. Data represented means±SD of 3 independent experiments. * t-test *P* value <0.05.

To examine the effect of fibroblast-sEVs on collagen metabolism, fibroblasts were incubated with medium containing SUI-sEVs, medium containing non SUI-sEVs and control medium respectively for 48 h. The Western blot showed that compared with control, type I and III collagen were decreased only in fibroblasts incubated with SUI-sEVs (Fig 2). Taken together, our data suggested that SUI-sEVs decreased the type I and III collagen expression in vaginal fibroblasts.

### SUI-sEVs enhanced the migration ability of fibroblasts

**Fig 3:**
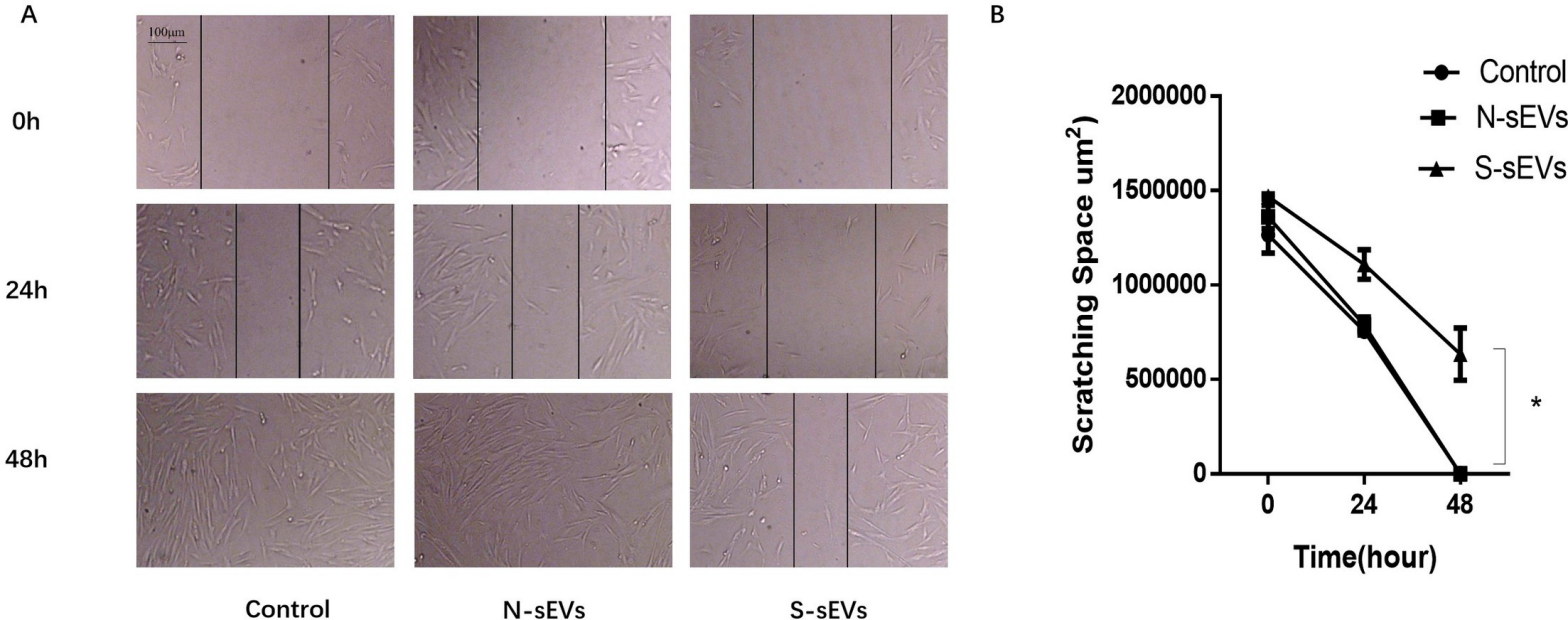
SUI-sEVs decreased the migration ability of primary cultured fibroblasts. (A) Fibroblasts were undergone scratch test, the wound healing changes were recorded between three groups at 0, 24 and 48h. (B) The variation of scratching space between groups is shown in the line chart. SUI-sEVs significant decreased the migration ability of primary cultured fibroblasts at 48h when compared to the cells in control group and treated with the non SUI-sEVs. N = 3 in each group. Data represented means±SD of 3 independent experiments. * t-test *P* value <0.05.

To examine the effect of fibroblast-sEVs on migration ability of fibroblasts, fibroblasts were incubated with medium containing SUI-sEVs, medium containing non SUI-sEVs and control medium respectively for 24h and 48 h in the scratch assay. The SUI-sEVs incubated cells were found to migrate significantly slower than the control and non SUI-sEVs group at 48 h after scratching (Fig 3).

### SUI-sEVs reduced the cell proliferation ability of fibroblasts

**Fig 4:**
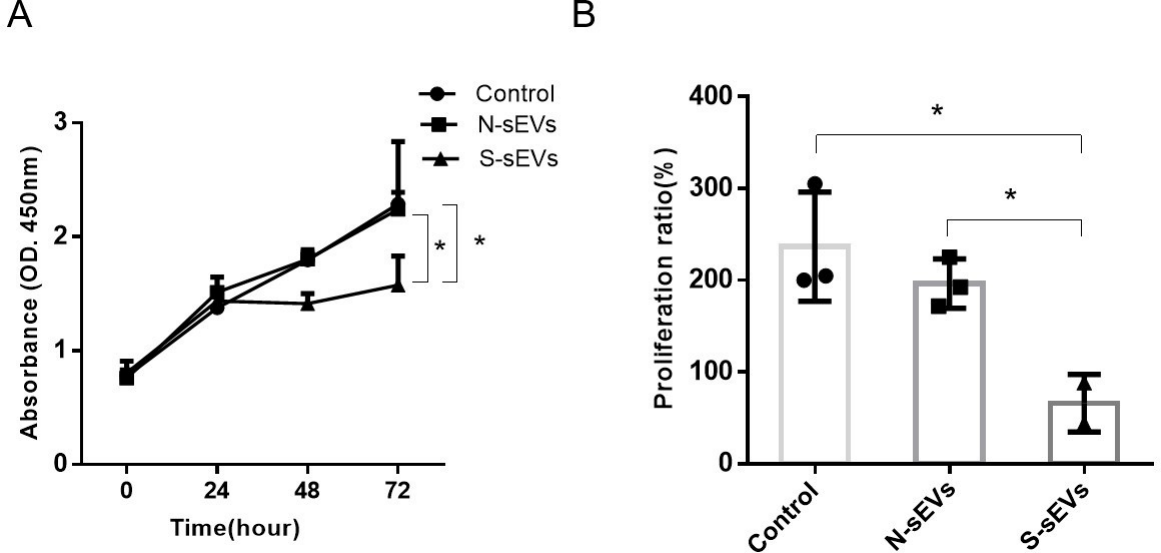
SUI-sEVs decreased the proliferation of fibroblasts. Fibroblasts were cultured with control medium or medium containing SUI-sEVs or medium containing non SUI-sEVs for 24, 48 and 72h, respectively. (A) Cell proliferation was determined with a CCK-8 assay. (B) The percentage of proliferative fibroblasts in three groups. SUI-sEVs significantly decreased the proliferation of fibroblasts after 72h when compared to the cells in control group and treated with the non SUI-sEVs. N = 3 in each group. Data represented means±SD of 3 independent experiments. * t-test *P* value <0.05.

Proliferation of fibroblasts under the stimulation of SUI-sEVs and non SUI-sEVs were evaluated by the CCK-8 test. The test showed that cell proliferation was obviously reduced in fibroblasts incubated with SUI-sEVs at 72h (Fig 4).

### Proteomic analysis of sEVs

**Fig 5:**
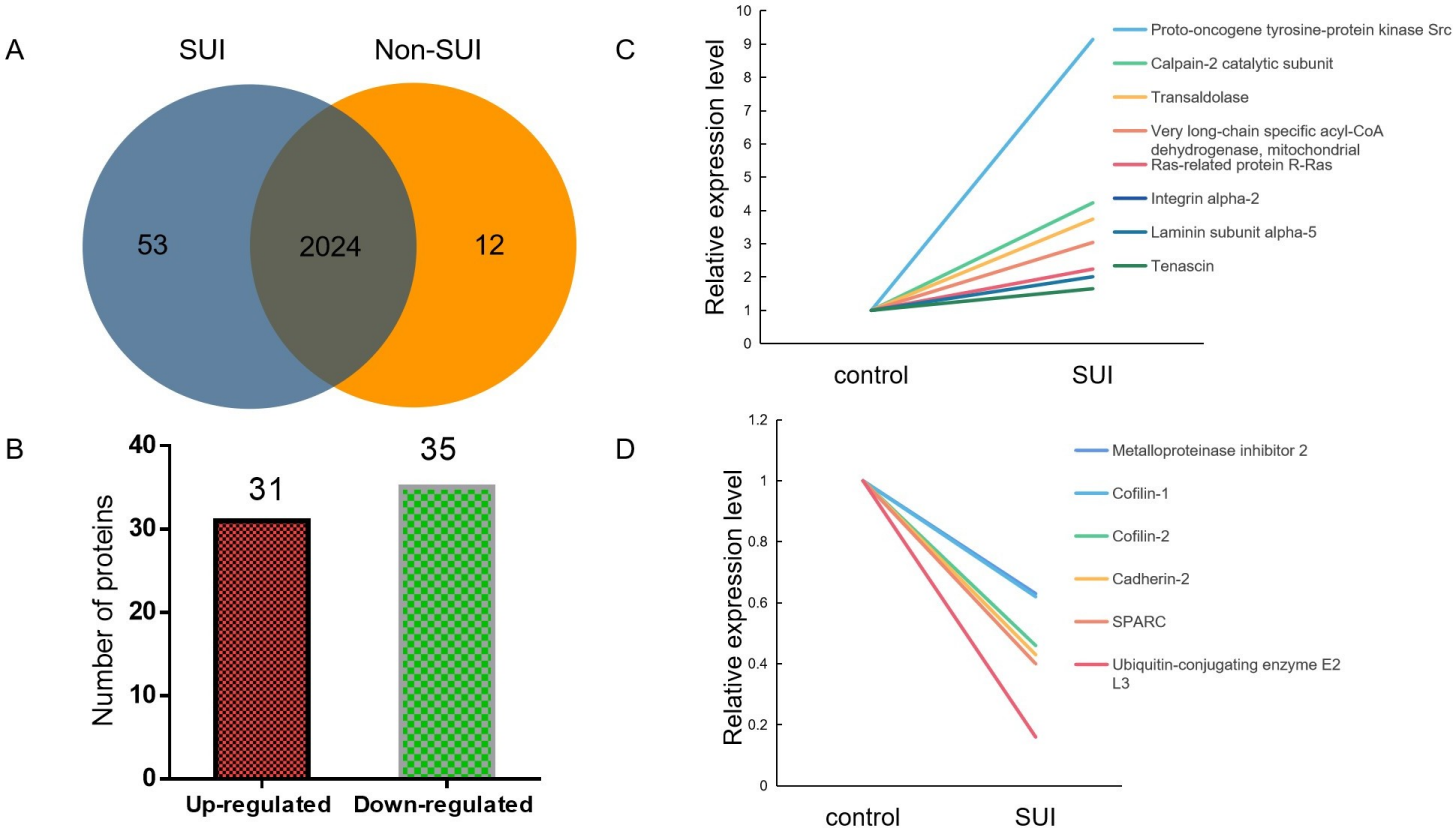
Summary of significant expressed proteins in fibroblast-sEVs of SUI patients or controls. (A) The number of identified proteins in each group was presented by Venn diagram. (B) Distribution of differentially regulated proteins. (C) Some significantly upregulated proteins in fibroblast-sEVs of SUI patients. (D) Some significantly downregulated proteins in fibroblast-sEVs of SUI patients. N = 3 in control group and N = 5 in SUI group.

Proteomic analysis of sEVs identified 2,089 proteins involved in various cell functions and pathways. Among these proteins, 53 protein was only found in SUI-sEVs and 12 proteins only in non SUI-sEVs (Fig 5A). To accurately evaluate the proteomic changes, more than1.2-fold change of the statistical value between two groups were defined as significantly expressed proteins. Compared with control, 31 proteins including proto-oncogene tyrosine-protein kinase Src, calpain-2 catalytic subunit, transaldolase, very long-chain specific acyl-CoA dehydrogenase, mitochondrial, ras-related protein R-Ras, integrin alpha-2, laminin subunit alpha-5, tenascin, were upregulated in SUI-sEVs (Fig 5B and 5C). And 35 proteins, including metalloproteinase inhibitor 2, cofilin-1, cofilin-2, cadherin-2, SPARC, ubiquitin-conjugating enzyme E2 L3 were downregulated in SUI-sEVs (Fig 5B and 5D). Part of significantly expressed proteins associated with the regulation of cell function and ECM were listed in the Table 1.

**Table 1:** Proteins differentially regulated in sEVs isolated from SUI patients.

Protein names	Gene names	Peptides	Unique peptides	P value	SUI VS N Ratio
Multidrug resistance-associated protein 4	ABCC4	15	15	0.035	21.69
Proto-oncogene tyrosine-protein kinase Src	SRC	8	5	0.035	9.14
Calpain-2 catalytic subunit	CAPN2	11	11	0.03	4.23
Transaldolase	TALDO1	6	6	0.035	3.74
Very long-chain specific acyl-CoA dehydrogenase, mitochondrial	ACADVL	10	10	0.035	3.04
Ras-related protein R-Ras	RRAS	10	8	0.03	2.24
Integrin alpha-2	ITGA2	35	35	0.03	2.09
Laminin subunit alpha-5	LAMA5	104	103	0.03	2.01
Tenascin	TNC	55	55	0.03	1.65
Metalloproteinase inhibitor 2	TIMP2	14	14	0.02	0.63
Cofilin-1	CFL1	11	8	0.03	0.62
Cofilin-2	CFL2	7	4	0.03	0.46
Cadherin-2	CDH2	7	7	0.03	0.43
SPARC	SPARC	13	13	0.03	0.40
Ubiquitin-conjugating enzyme E2 L3	UBE2L3	3	3	0.0285	0.16

Statistical analyses were performed by Chi-square test.

*P* < 0.05 was considered significant.

### GO and KEGG pathway analysis

**Fig 6:**
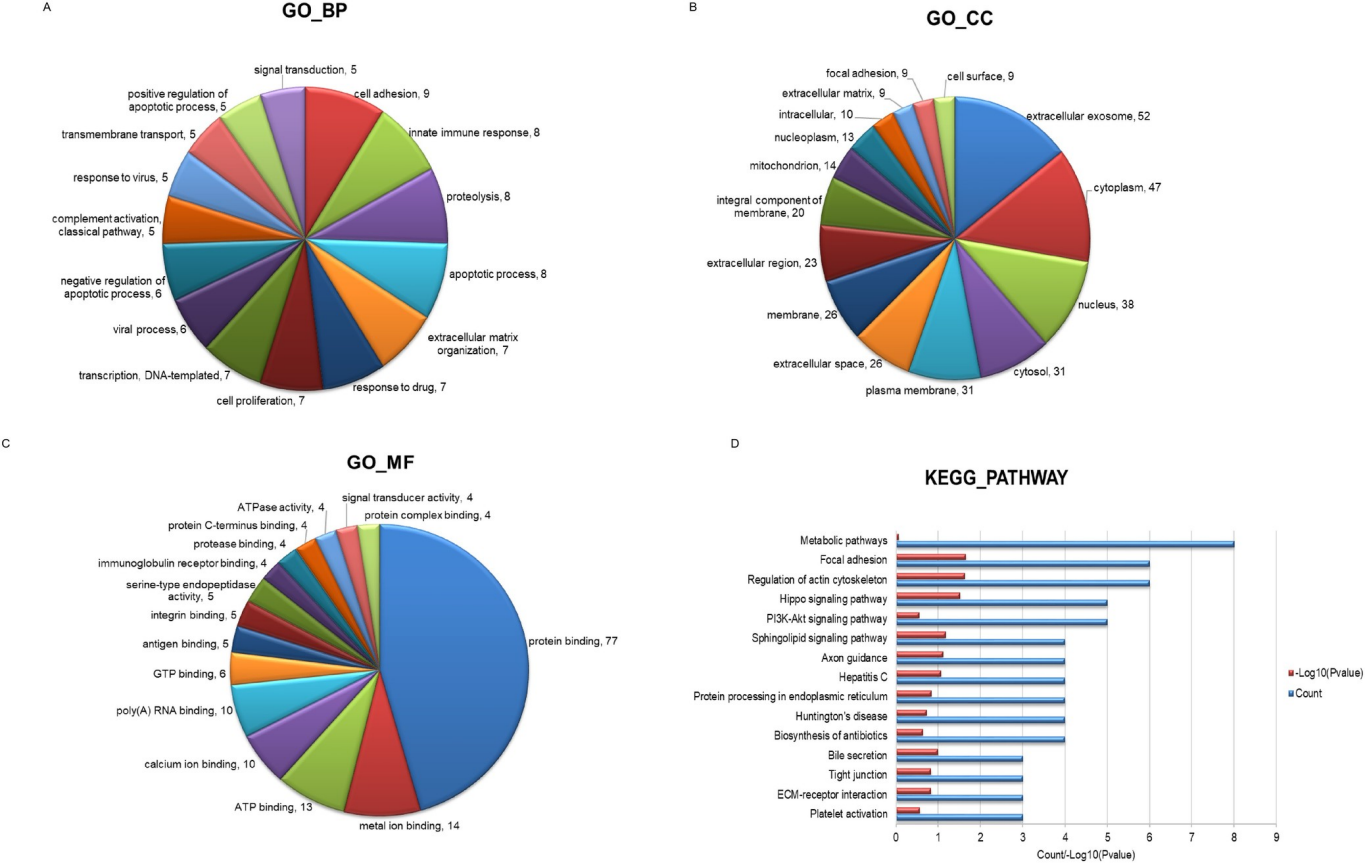
The Bioinformatics analysis of the proteins carried by the sEVs. (A-C) GO functional classification of fibroblast-sEVs proteomes. (A) Biological process, (B) cellular component, and (C) molecular function. The numbers after comma indicate the count of the proteins. (D) The KEGG analysis of the sEVs.

The GO and KEGG pathway analysis were implemented to further evaluate the result of proteomic analysis of fibroblast-sEVs. The results of GO analysis showed that 207 proteins were associated with specific biological processes, 77 with diverse cellular components, and 72 with other molecular functions. Graphs showed the relationship between each of the three ontologies and the number of the associated proteins (Fig 6A–6C). The results of KEGG pathway analysis showed that 57 different pathways were linked to the sEVs. The top 15 KEGG pathways were shown in Fig 6D, and proteins of pathways related to extracellular matrix remodeling and fibroblast function were shown in Table 2.

**Table 2:** Pathway analysis of fibroblast-sEVs involved in SUI compared with control.

Pathway	Gene Symbol
Metabolic pathways	ACADVL↑, SDHB↑, ATP5J2↑, TALDO1↑, ALDH5A1↑, PAPSS1↑, GART↑, PYGB↑
Focal adhesion	LAMA5↑, TNC↑, ITGA2↑, CAPN2↑, SRC↑, MYLK↑
Regulation of actin cytoskeleton	CFL2↓, CFL1↓, RRAS↑, ITGA2↑, SRC↑, MYLK↑
PI3K-Akt signaling pathway	LAMA5↑, PPP2CA↑, TNC↑, PPP2CB↑, ITGA2↑
ECM-receptor interaction	LAMA5↑, TNC↑, ITGA2↑
Endocytosis	AP2B1↑, RAB22A↑, SRC↑
Apoptosis	BAX↑, CAPN2↑
TGF-beta signaling pathway	PPP2CA↑, PPP2CB↑
AMPK signaling pathway	PPP2CA↑, PPP2CB↑
Oxidative phosphorylation	SDHB↑, ATP5J2↑
Ubiquitin mediated proteolysis	SOCS3↓, UBE2L3↑
Cell adhesion molecules (CAMs)	LRRC4↑, CDH2↓
Phagosome	TUBB↑, ITGA2↑
Purine metabolism	PAPSS1↑, GART↑
cAMP signaling pathway	RRAS↑, ABCC4↑
Rap1 signaling pathway	RRAS↑, SRC↑

## Discussions

SUI is a common disease in female. ECM, which provided structure and biochemical support to the nearby cells, was regulated by fibroblasts. Imbalance of ECM remodeling in the anterior vaginal wall was reported to be involved in the pathogenesis of SUI [3, 23]. In this study, we investigate the effects of sEVs secreted by vaginal fibroblasts on the pathogenesis of SUI. Our results demonstrated that over-expression of fibroblast-sEVs from SUI patients inhibited the collagen synthesis, proliferation and migration capacity of fibroblasts. And, the differentially expressed proteins including TIMP2, TGF-β and ABCC4 in fibroblast-sEVs may be involve in pathogenesis of SUI.

The sEVs could deliver proteins, lipids, genetic materials and other active substances to participate in biological processes [24]. It had been proven that sEVs were involved in various physiological and pathological processes [24]. Responses mediated by sEVs could be disease-promoting, indicating potential utility in the diseases control [24]. It was reported that after exposing to gemcitabine, cancer-associated fibroblasts significantly increased the release of sEVs, which further regulated proliferation of pancreatic cancer cells and drug resistance [14]. Furthermore, several studies showed that cardiac fibroblasts secreted miRNA-enriched sEVs, which played as a paracrine signaling mediator of cardiomyocyte hypertrophy [25]. However, the effects of fibroblast-sEVs on the pathogenesis of SUI remained unclear. In the present study, we successfully isolated and purified sEVs from human fibroblasts of paraurethral anterior vaginal wall tissue. We further showed fibroblast-sEVs from SUI patients were significantly higher than those from non-SUI patients. The finding suggested that fibroblast-sEVs may be involved in the pathogenesis of SUI.

The dysfunction of fibroblasts was crucial in development of SUI. It was reported that collagen I and III were reduced in fibroblasts from the SUI patients, and elevated collagen I and III in fibroblasts relieved symptoms of SUI [4, 26]. In addition, by regulating proliferation, migration, apoptosis, differentiation and other functions, fibroblasts participated in pathogenesis of various diseases [27–30]. Recently, it was found that human circulating fibrocytes stimulated by PDGF BB (Platelet-derived growth factor-BB) and TGF - β1 (Transforming growth factor-β1) promoted proliferation and migration, and increased collagen I and III synthesis of human dermal fibroblasts [15]. Also, Liu et al showed that sEVs secreted by mesenchymal stem cells could increase synthesis and reduce degradation of collagen in vaginal fibroblasts from SUI [31]. Based on the above studies, we co-cultured fibroblasts with SUI-sEVs and non SUI-sEVs, and the results showed SUI-sEVs reduced the collagen synthesis, proliferation and migration capacity of fibroblasts. This revealed a possible mechanism by which fibroblast-sEVs involved in the pathogenesis of SUI.

Exact role of fibroblast-sEVs in the regulation of SUI needs further study. In our study, we found fibroblast-sEVs contained various proteins involved in a variety of pathways by GO and KEGG pathway analysis. Some of these proteins were associated with PI3K-Akt and TGF-β signaling pathways that were related to proliferation and activation of fibroblasts [32, 33]. For instance, ABCC4 (aka MRP4) was a member of the ATP-binding cassette family of membrane transporters, which regulated multiple cyclic nucleotide-dependent cellular events including cell migration. ABCC4 was found to play key roles in the fine-tuned regulation of fibroblast migration by modulating intracellular cyclic nucleotide concentration [34]. It was reported that SRC contributed to cell growth, proliferation, adhesion, ECM expression and migration [35, 36]. Previous studied found that fibroblasts treated with the selective inhibitor of SRC had a decreased expression of collagen I [37]. In addition, it was reported that calpain (CAPN), as an intracellular calcium-dependent neutral cysteine endopeptidase, exerted significant impacts on ECM remodeling. CAPN2, as a subtype of CAPN, played a crucial role in the expression of hepatic fibrosis markers, including COL3A1, COL1A1 and MAPK1 [38]. And siRNA knockdowns of CAPN2 impaired cell invasion and MMP secretion [39]. Therefore, these results support that the proteins in sEVs may affect the function of fibroblasts by stimulating related signaling pathways.

However, there were also some limitations in this study. Firstly, the sample size of our study was limited and needed further research with large sample size. Secondly, functional validation of certain identified proteins in vitro was needed in the future work.

## Conclusions

In conclusion, our study showed human vaginal fibroblast-sEVs were overexpressed in SUI patients, suggesting that fibroblast-sEVs might be involved in the pathogenesis of SUI. Studies of co-culturing sEVs and fibroblasts further relvealed over-expression of fibroblast-sEVs from SUI patients significantly inhibited the collagen synthesis, proliferation and migration capacity of fibroblasts, indicating fibroblast-sEVs might play a role in the onset of ECM related SUI. Mass spectrometric analysis revealed the differentially expressed proteins including TIMP2, TGF-βand ABCC4 in fibroblast-sEVs might be related to the potential mechanism. In all, fibroblast-sEVs exerted inhibitory effect on the function of fibroblasts, which participated in the pathogenesis of ECM related SUI. Further research focusing on specific proteins might innovate new strategy to prevent or mitigate SUI.

## Supporting information

S1 Table Supplemental table of the peptide identifications. (XLSX)

S2 Table Supplemental table of the protein identifications. (XLSX)

S1 Raw images (PDF)
