# Supplementary figures and images for: Small extracellular vesicles secreted by vaginal fibroblasts exert inhibitory effect in female stress urinary incontinence through regulating the function of fibroblasts
Source: PLoS One. 2021 Apr 9;16(4):e0249977. doi: 10.1371/journal.pone.0249977 (PMC8034718; doi:10.1371/journal.pone.0249977)

## S1 Raw images

**Figure 2A**

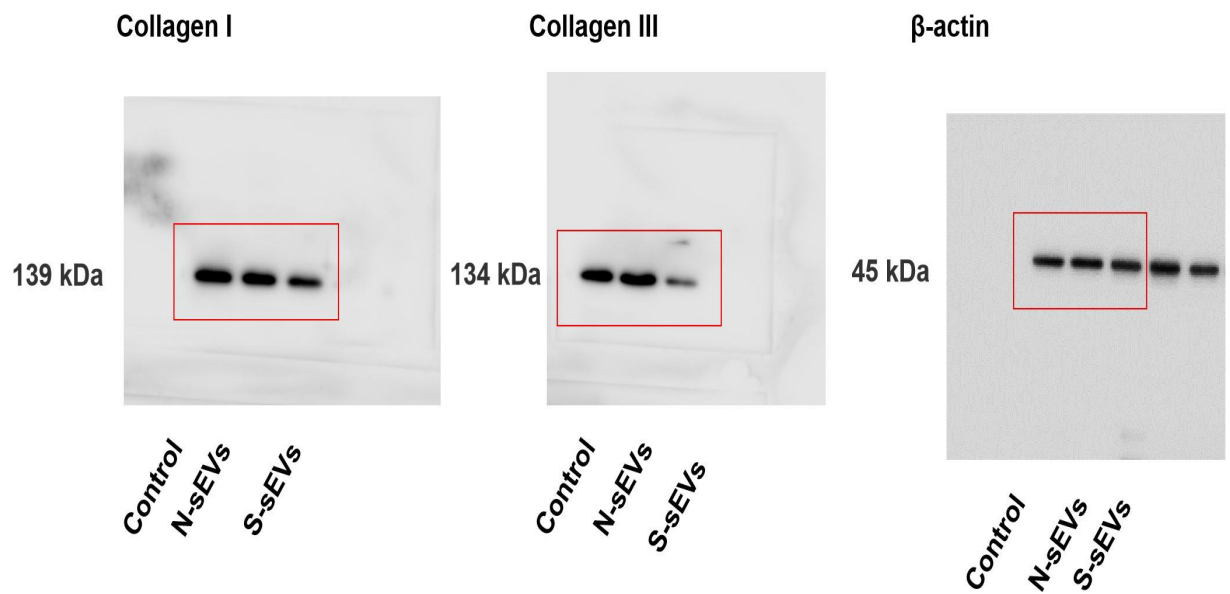

Supplement: S1 Raw images — (PDF) [file pone.0249977.s003.pdf]
